# Effect of resveratrol and metformin on ovarian reserve and ultrastructure in PCOS: an experimental study

**DOI:** 10.1186/s13048-018-0427-7

**Published:** 2018-06-29

**Authors:** Selenay Furat Rencber, Sema Kurnaz Ozbek, Ceyla Eraldemır, Zehra Sezer, Tugba Kum, Sureyya Ceylan, Elif Guzel

**Affiliations:** 1 Department of Histology and Embryology, Kocaeli University Faculty of Medicine, 41380 Kocaeli, Turkey; 2 Department of Biochemistry, Kocaeli University Faculty of Medicine, 41380 Kocaeli, Turkey; 3 Department of Histology and Embryology, Istanbul University-Cerrahpasa Faculty of Medicine, 34098 Istanbul, Turkey

**Keywords:** Dehydroepiandrosterone, Resveratrol, Metformin, Polycystic ovary syndrome, Oxidative stress, Inflammation

## Abstract

**Background:**

PCOS is a reproductive hormonal abnormality and a metabolic disorder. It is frequently associated with insulin resistance, hyperandrogenism, chronic inflammation, and oxidative stress. We aim to investigate the potential therapeutic effects of combined therapy of resveratrol and metformin on polycystic ovaries via SIRT1 and AMPK activation.

**Methods:**

Wistar albino rats were divided into control and experimental (PCOS) groups. DHEA-induced PCOS rats were given resveratrol (20 mg/kg/day), metformin (300 mg/kg/day) and combined therapy. At the end of the experiment, the body and ovarian weight of rats were measured and blood samples were analyzed for FSH, LH, testosterone, AMH, TNF-α and MDA levels. Histopathological evaluation of ovaries were carried out by light and electron microscopy. SIRT1 and AMPK immunreactivity and TUNEL assay were scored. Data were statistically analyzed by SPSS programme.

**Results:**

Metformin and combined treatment groups reduced the body and ovary weights compared to the PCOS group. Serum testosterone levels were significantly higher in the PCOS group than in the control group and this was reduced when PCOS was treated with all but especially resveratrol. All the treatment groups decreased LH, LH/FSH, TNF-α and tissue AMH levels which were induced in the PCOS group, whereas metformin was unable to improve the increased MDA and plasma AMH levels. Treatment with resveratrol and/or metformin ameliorated the elevated number of secondary and atretic follicles and the decreased number of Graafian follicles in the PCOS group, which indicates the effect of the treatments on the maintenance of folliculogenesis. Light and electron microscopic findings supported the analysis of follicular count. Increased number of TUNEL (+) granulosa cells in the PCOS group were reduced significantly in the treatment groups. Resveratrol and metformin increased SIRT1 and AMPK immunreactivity, respectively, compared to the PCOS group.

**Conclusions:**

The results suggest that combined therapy of metformin and resveratrol may improve the weight gain, hormone profile and ovarian follicular cell architecture by inducing antioxidant and antiinflammatory systems via SIRT1 and AMPK activation in PCOS.

## Background

Polycystic ovary syndrome (PCOS) is a reproductive hormonal abnormality and a systemic-metabolic disorder. This syndrome affects an estimated 6–21% of reproductive-age women and is one of the main cause of infertility [1]. Although the pathogenesis mechanism has not been well defined, PCOS is frequently associated with insulin resistance, chronic inflammation, and oxidative stress (OS) [2, 3]. Increased secretion of LH compared to FSH and hyperandrogenism are also classical features of PCOS that 70–80% of women with hyperandrogenism are diagnosed with PCOS [4, 5]. Hyperandrogenism is correlated wıth inflammation in PCOS pathogenesis [6]. Furthermore, recent studies demonstrated that inflammatory markers, such as C-reactive protein (CRP), tumor necrosis factor (TNF), interleukin-6 (IL-6), interleukin-18 (IL-18), monocyte chemotactic protein-1 (MCP-1), and acute phase serum amyloid A (APSAA) increase in women with PCOS [7–10].

Classical morphology of PCOS includes ovarian cortical thickening, multiple tiny capsular follicular cysts, luteinized inner theca, stromal hyperplasia and multiple immature follicles, which indicate cessation of folliculogenesis [11]. Anti-Mullerian hormone (AMH) is secreted by the granulosa cells of secondary follicles and has an important role in both ovarian primordial follicle recruitment and dominant follicle selection [12]. Since it has been suggested that there is a correlation between antral follicle count and AMH, this hormone has been proposed as a marker for PCOS [13].

Resveratrol (3,5,4-trihydroxystilbene) is a natural polyphenol, found in grapes, red wine, peanuts, and several medicinal plants [14]. This agent may have antidiabetic, antioxidant and antiinflammatory actions [15–17]. Depending on the cell type, resveratrol has been shown to induce both proapoptotic [18, 19] and antiapoptotic [20, 21] effects in in vitro and in vivo studies. Kong et al. suggested that resveratrol significantly increases the total number of oocytes, significantly decreases the atretic follicles and inhibits both the primordial-to-developing-follicle transition and apoptosis in different age groups of rats [22]. In a recent study in rodents, resveratrol protected oocytes from methylglyoxal-induced cytotoxicity, and this effect was mediated by decreasing reactive oxygen species (ROS) production [23]. However, at present, there is a paucity of information about potential beneficial actions of resveratrol on reproductive functions and ovary.

Resveratrol interacts with multiple cellular targets, but many effects of resveratrol are attributed to its activation of SIRT1 (silent information regulator 1) [24]. SIRT1, the mammalian homologue of yeast Sir2, deacetylates several targets in mammalian cells, acting as a key regulator of energy homeostasis, gene silencing, metabolism, genomic stability, and cell survival [24–26]. In ovary, SIRT1 is expressed in oocytes and nuclei of human granulosa cells at various stages of follicular development and known to exhibit suppressive effects on inflammation in various cell types. Besides, it is suggested that it defends oocytes from age-dependent deficits through cellular protection against OS [27].

Metformin (1,1-dimethylbiguanide hydrochloride), the insulin sensitizer, is an oral antihyperglycemic drug widely used for the treatment of type 2 diabetes [28] and PCOS [29]. Metformin can cause reductions in body weight [30] and restore ovulation [31]. It is also suggested that metformin reduces androgen synthesis from ovarian theca cells and suppresses ovarian steroidogenesis [32, 33]. Generally, its mechanism of action is through the activation of the AMP activated kinase (AMPK), which acts as an energy sensor by monitoring the AMP/ATP status of the cell [34, 35]. Interestingly, many target proteins of AMPK are so-called longevity factors, e.g., SIRT1, p53, and FoxOs, which not only can increase the stress resistance and extend the lifespan of many organisms but also inhibit the inflammatory responses [36]. Moreover, AMPK activation protects against arachidonic acid+iron-induced OS through the inhibition of mitochondrial impairment and ROS production [37]. However, the exact mechanism by which metformin improves ovarian function remains unclear.

Although severe adverse effects are rare, up to 30% of patients report gastrointestinal symptoms, including diarrhea, cramps, nausea, and vomiting, which can cause severe discomfort and lead to discontinuation of the drug [38]. Finding alternative strategies better than or at least comparable with metformin’s efficacy for the treatment of PCOS is a useful approach for managing the adverse events of metformin. Since resveratrol, as an antioxidant and antiinflammatory agent is known to have some beneficial effects on the ovary and with the given information above about the AMPK-SIRT1 pathway [27, 36, 37], in this study we hypothesise that combined therapy of resveratrol and metformin might have beneficial effects on PCOS via SIRT1 and AMPK activation. To test this hypothesis, in addition to the detection of TNF-α, MDA and serum hormone levels to measure inflammation, OS and hyperandrogenism, respectively, we investigated the number of follicles, the apoptotic index of the follicular cells, SIRT1 and AMPK immunreactivity in the follicles and the ultrastructure of the oocytes and the granulosa-theca cells in an experimental PCOS model. Because PCOS is the most common endocrine-metabolic disorder and causes infertility among women, focusing on the resveratrol and metformin’s potential therapeutic effects on ovary will be useful for public’s health.

## Methods

### Animal maintenance and treatment

The experiments were conducted in accordance with the Regulation of Animal Research Ethics in Turkey. The ethical approval was granted by the Kocaeli University Animal Research Ethics Committee (Project number 2015/8, Kocaeli, Turkey). All experiments were conducted between 9:00 a.m. and 12:00 p.m. under standard laboratory conditions (22 ± 2°C room temperature; 12-h light/dark cycle with lights on at 7:00 a.m.). Tap water and food pellets were provided ad libitum.

Sixty-three immature female Wistar albino rats (three weeks-old, weighing 45–50 g) were initially divided into control (*n* = 9) and experimental (PCOS) groups (*n* = 54). Control group received 0,2 ml sesame oil subcutaneously (s.c.). PCOS was induced by administration of 6 mg/100 g body weight dehydroepiandrosterone (DHEA: sc-202,573; Santa Cruz Biotechnology, Inc., CA) s.c. dissolved in 0.2 ml sesame oil in experimental group. The daily treatments of both groups lasted for up to 35 days consecutively [39]. After modelling, experimental (PCOS) group (n = 54) were randomly divided into 6 groups (n = 9) as follows: Group 1 (PCOS; no treatment), Group 2 (Resveratrol; 20 mg/kg resveratrol [22]. (sc-200,808; Santa Cruz Biotechnology, Inc., CA) dissolved in %5 ethanol- i.p.), Group 3 (%5 ethanol; resveratrol solvent- i.p. [40]), Group 4 (Metformin; 300 mg/kg metformin (sc-202,000; Santa Cruz Biotechnology, Inc., CA), dissolved in 1.0 ml saline- oral gavage [39].), Group 5 (1.0 ml saline; metformin solvent- oral gavage) and Group 6 (Metformin+Resveratrol; 20 mg/kg resveratrol and 300 mg/kg metformin). Further treatments for the PCOS groups lasted for 28 days. The dose of metformin administered was equivalent to the dose administered to human patients [41].

### Vaginal cytology

Smears were performed on control and experimental group during last 12 days of modeling and stained with Papanicolau. Rats’ estrous cyclicity was monitored by vaginal smears and assessed by light microscopy.

### Body and ovary weights

Rats were weighed every day to calculate drug doses and at the end of the experiment, body and the excised ovary weights were measured.

### Tissue sample collection

At the end of the experiment, all rats were anesthetized (2.5 mg/kg Alfazyne and 12.5 mg/kg Ketalar) and blood samples were obtained by cardiac puncture. Sera and plasma were immediately separated and kept at − 20 °C for subsequent analyses. Following the collection of blood samples, both ovaries of rats were quickly removed and cleaned. Left ovaries were fixed in 10% neutral buffered formalin solution for light microscopic tissue processing whereas a half of the right ovaries were fixed in 2.5% gluteraldehide for ultrastructural assessment by electron microscopy. Other half of right ovaries were quickly frozen at − 40 °C for biochemical analyses.

### Biochemical analyses

#### Tissue homogenization

The tissues were homogenized with a tissue homogenizer in PBS (1/10; weight/volume) [42]. Then, the samples were centrifuged and the supernatant was collected for analyses.

#### Determination of total protein in tissue samples

The total protein content of tissue samples was quantified by modified Lowry’s method [43]. Enzyme-linked immunosorbent assay (ELISA) results were given as a percentage of the total protein concentration of the sample.

#### Tissue and plasma TNF-α and AMH levels

Tissue and plasma AMH and TNF-α levels were determined by ELISA method (Bioassay; Shanghai, China) according to the manufacturer’s instructions.

#### Serum hormone levels

Serum FSH, LH, and testosterone levels were measured by autoanalyzer (Beckman Coulter DXI-600).

#### Serum MDA levels

The substrat of lipid peroxidation, MDA, reacts with thiobarbirutic acid and gets pink colour complex at 535 nm absorbance. The colour volume of this complex was measured spectrophotometrically and MDA levels of the sample were determined [44, 45].

### Light microscopic examinations

The left ovarian tissues were dehydrated with ethanol and toluene, then embedded in paraffin. 4–5 μm-sections were stained with hematoxylin and eosin (H&E) to determine follicle count and ovarian architecture. Sections from the center of all ovaries were applied to AMPK, SIRT1 and TUNEL method to examine immunohistochemistry.

#### Histology and follicle count

To evaluate ovarian architecture and follicle number, hematoxylin-eosin staining were applied on every 10th section of the ovary so that each section was separated by a distance of approximately 50–60 μm from the next 10th section [46]. Follicles were counted according to the following definitions: Primordial follicle; surrounded by thin, single layers of the so-called follicular epithelial cells. Primary follicle; uni- or multilaminar follicular epithelium that surrounds the oocyte becomes iso- to highly prismatic. Secondary follicle; follicle in which oocyte was covered with more than two layers of granulosa cells and in which antrum formation commenced. Graafian (tertiary) follicle; follicle that possesses a single and large antrum filled with follicular fluid, in which granulosa cells surround the antrum, and that oocyte surrounded by some granulosa cells (cumulus cells) [47]. Atretic follicle; degenerated zona pellusida or oocyte along with pyknosis in granulosa cells; granulosa cells and cell debris within antral cavity [46, 48]. All sections were evaluated with an optical microscope (Leica DM 1000) and monitored with attached digital camera (Leica DMC 2900).

#### SIRT1 and AMPK immunohistochemistry

A section taken from the central level of the ovary from each group was chosen to determine SIRT1 immunoreactivity. Specimens were deparaffinized in fresh toluene (3 × 5 min), then treated with graded series of ethanol for rehydration (100, 100, 95, and 80%; 5 min each) and rinsed with tap water, followed by two rinses with distilled water (1–2 min each). To perform heat-induced antigen retrieval, the slides were kept in citrate buffer (pH = 6,0) for 1 × 10 min. Endogenous peroxidase activity was inhibited in 3% hydrogen peroxide (H_2_O_2_) (Merck, Darmstadt, Germany) then, the sections were washed in phosphate-buffered saline (PBS, pH = 7.6) containing 0.1% Triton X (T-PBS) twice. The epitopes were stabilized by protein block solution (SHP125; ScyTek, West Logan, Utah). After removing excess serum, 50 μL of polyclonal SIRT1 (H-300: sc-15,404; Santa Cruz Biotechnology, Inc., CA) primary antibody (dilution factor 1:100) was added to each specimen which were then incubated in a humidified chamber at 37 °C for 1 h [49]. After rinsing with PBS (3 × 5 min), biotinylated secondary antibody (SHP125; ScyTek, West Logan, Utah) was applied for 20 min. Afterward, streptavidin-peroxidase solution (SHP125; ScyTek, West Logan, Utah) was applied to the slides, then 3,3′-diamino benzidine (DAB) solution (ACH500; ScyTek, West Logan, Utah) or 3-amino-9-ethylcarbazole (AEC) solution (ACJ500; ScyTek, West Logan, Utah) was performed as a chromogen. Mayer’s hematoxylin was used for counterstaining. The specimens were mounted with Entellan (Merck Milipore, Darmstadt, Germany) for microscopic examination.

AMPK immunohistochemistry procedure was same with SIRT1 except for antibody dilution and incubation time. AMPKα1/2 primary antibody (H-300: sc-25,792; Santa Cruz Biotechnology, Inc., CA) dilution factor was 1:1000 and incubation time were 24 h according to the instructions. [50].

SIRT1 and AMPK expression intensity of follicular cells were measured by a histological score (HSCORE) method. 0, no staining; 1, weak staining; 2, moderate staining; 3, intense staining [51]. For each slide, HSCORE value was derived by summing the percentage of cells that stained at each intensity category and multiplying that value by the weighted intensity of the staining, using the formula HSCORE = ∑Pi (i + 1), where i is the intensity score, and Pi is the percentage of labeled cells for each intensity within a range of 0–100%.

#### Terminal deoxynucleotidyl transferase-mediated dUTP nick end labeling (TUNEL) assay

TUNEL assay was used for detection of DNA damage. Assay procedure was performed following the manufacturer’s instructions (S7101; Apoptag Plus Peroxidase In Situ Apoptosis Kit, Merck Millipore, Darmstadt, Germany). Briefly, sections taken from the central level of the ovary from each group were dewaxed in toluene and rehydrated through a graded series of ethanol and double-distilled water. Slides were rinsed with PBS and kept in proteinase K (Sigma–Aldrich, St. Louis, MO) for antigenic retrieval. The sections were incubated with 3% H_2_O_2_ (Merck, Darmstadt, Germany) for 5 min, rinsed and then incubated with 1× equilibration buffer at room temperature for 10 min. TdT (terminal deoxynucleotidyl transferase) reaction solution was applied at 37 °C for 1 h, except that distilled water was used instead of TdT enzyme as a negative control. Sections were subsequently put into stop/wash buffer at room temperature for 10 min. Antidigoxigenin-peroxidase conjugate was applied to the sections. After applying DAB as a chromogen, sections were counterstained with methyl green (TM of Trevigen). In all type of follicles, the percentage of TUNEL (+) cells to total cell number was recorded as apoptotic index [52].

### Electron microscopic examination

Half of the left ovaries were cut into 1 mm^3^ strips, fixed with 2.5% gluteraldehyde, rinsed in Millonig’s buffer (pH 7.4) and then postfixed in 1% osmium tetroxide. The samples were dehydrated in graded series of ethanol, kept in propylene oxide and embedded in araldite (Sigma–Aldrich, St. Louis, MO). Semi-thin sections from the polymerized blocks were stained with methylene blue and screened by light microscopy for the presence of follicles. Ultra-thin sections (Reichert UM3 ultramicrotome, 50–60 nm) were contrasted with uranyl acetate and lead citrate and then analyzed by a transmission electron microscope (Jeol-1011, Tokyo, Japan). Ultrasutructure of follicular cells and oocytes were evaluated. In granulosa and theca cells; we evaluated the cytoplasmic membrane and the density and integrity of the mitochondrial cristae, smooth endoplasmic reticulum (sER), lysosomes, and lipid droplets. In oocyte, the fracture or delamination of the zona pellucida, enlarged perivitelline space and oocyte-granulosa cell connections were examined [53, 54].

### Statistical analyses

The statistical analysis was performed using IBM SPSS 20.0 (SPSS Inc., Chicago, IL, USA). All data was expressed as the mean ± standard deviation (SD). Kolmogorov-Smirnov test was used to assess the normality of the data. Comparisons of multiple samples were performed using either one-way ANOVA with post-hoc Tukey test or Kruskal-Wallis test with Dunn’s post-hoc test. Tests were performed within 95% confidence interval and significant level was based on *p* < 0.05.

## Results

### Estrus Cyclicity determination

Vaginal cytology of the DHEA-administered rats exhibited irregular estrous cyclicity, thus PCOS modeling was successfully established.

### Body and organ weights

The data of body and ovary weight are shown in Table 1. Body weights of the rats in the PCOS group were significantly higher than in the control group (*p* < 0.001). Metformin and combined treatments reduced the body weights compared to the PCOS group (*p* < 0.05). Although resveratrol had reducing effects on weight change, it wasn’t statistically significant. Also, no significant difference was observed between resveratrol and other treatment groups. Ovarian weights were increased significantly in the PCOS group when compared to the control group (*p* < 0.001) and it was significantly decreased in all treatment groups compared to the PCOS group (*p* < 0,001). When treatment groups were compared within themselves, the only significant difference were observed between resveratrol and the other treatment groups (*p* < 0.001).

**Table 1:** Mean ± SEM of body and ovary weight in control, PCOS, resveratrol, metformin and metformin+resveratrol treated rats

Parameters	Body Weight (g)	Ovary Weight (g)
Control	154.33 ± 2.13	0.06 ± 0.002
PCOS	180.44 ± 2.02^**^	0.08 ± 0.001^**^
Resveratrol	172.89 ± 3.71^*^	0.07 ± 0.001^++,**^
Metformin	159.33 ± 1.39^+^	0.06 ± 0.001^++,##^
Metformin+Resveratrol	159.89 ± 1.90^+^	0.07 ± 0.001^++, *,##^
%5 Ethanol (resveratrol solvent)	176.71 ± 1.82^**^	0.08 ± 0.001^**,#^
Saline (metformin solvent)	178.00 ± 3.22^**^	0.08 ± 0.002^**,##^

Values are expressed as mean ± SEM

*, ** versus control group; *P* < 0,05 and *P* < 0.001, respectively

^+^, ^++^ versus PCOS group; P < 0,05 and P < 0.001, respectively

^#^, ^##^ versus resveratrol group; P < 0,05 and P < 0.001, respectively

### Biochemical parameters

Table 2 shows the data of the biochemical parameters.

**Table 2:** Mean ± SEM of FSH, LH, LH/FSH, testosterone, plasma and tissue AMH, plasma and tissue TNF-α and serum MDA levels in control, PCOS, resveratrol, metformin and metformin+resveratrol treated rats

Parameters	Control	PCOS	Resveratrol	Metformin	Metformin+Resveratrol	%5 Ethanol	Saline
Serum FSH (mIU/ml)	0.09 ± 0.01	0.13 ± 0.01	0.14 ± 0.01	0.1 ± 0.01	0.1 ± 0.01	0.14 ± 0.02	0.13 ± 0.01
Serum LH (mIU/ml)	0.03 ± 0	0.11 ± 0.01^**^	0.04 ± 0.01^++^	0.03 ± 0.01^++^	0.03 ± 0.01^++^	0.12 ± 0.01^**^	0.1 ± 0.01^**^
LH/FSH	0.33 ± 0.07	0.82 ± 0.08^*^	0.30 ± 0.05^+^	0.39 ± 0.11^+^	0.42 ± 0.1^+^	0.87 ± 0.08^*^	0.89 ± 0.13^*^
Testosterone (ng/ml)	0.84 ± 0.02	1.91 ± 0.09^**^	1.04 ± 0.03^++^	1.21 ± 0.03^**,++^	1.13 ± 0.04^*,++^	1.3 ± 0.06^**^	1.27 ± 0.05^**^
Plasma AMH (ng/ml)	3.19 ± 0.1	5.1 ± 0.24^*^	3.94 ± 0.16^+^	4.57 ± 0.25^*^	3.93 ± 0.17^+^	–	3.94 ± 0.25
Tissue AMH (ng/10 mg prot.)	5.92 ± 0.16	9.14 ± 0.66^*^	6.17 ± 0.26^++^	6.79 ± 0.35^++^	6.18 ± 0.32^++^	–	6.44 ± 0.4
Plasma TNF-α (ng/ml)	176.48 ± 5.53	276.59 ± 19.28^*^	195.46 ± 15.66^+^	206.22 ± 19.68^+^	193.88 ± 13.51^+^	–	250.42 ± 6.6
Tissue TNF-α (ng/10 mg prot.)	25.86 ± 0.46	36.1 ± 2.18^**^	28.64 ± 0.54^+^	28.8 ± 0.33^+^	27.95 ± 1.52^+^	–	32.04 ± 1.76
Serum MDA (ng/L)	0.9 ± 0.1	1.8 ± 0.22^*^	1 ± 0.11^+^	1.19 ± 0.19	0.98 ± 0.21^+^	–	1.41 ± 0.17

Values are expressed as mean ± SEM

*, ** versus control group; P < 0,05 and P < 0.001, respectively

+, ++ versus PCOS group; P < 0,05 and P < 0.001, respectively

#### Serum, plasma and tissue hormone levels

There were no significant difference between groups in terms of FSH levels. Serum LH levels and LH/FSH ratio were significantly higher in the PCOS group compared to the control group (*p* < 0,001 and p < 0,05, respectively) and these parameters in PCOS group were significantly reduced by all treatments (*p* < 0,001 and *p* < 0,05, respectively). No significant difference was observed between treatment groups.

Because of the excess androgen accumulation, serum testosterone levels were significantly higher in the PCOS group compared to the control group (*p* < 0,05). All treatment groups, especially the resveratrol group significantly reduced this hormone levels when compared to the PCOS group (*p* < 0,05). No significant difference was observed between treatment groups.

Plasma and tissue AMH levels were significantly higher in the PCOS group than in the control group (*p* < 0,05), whereas the same hormone levels were significantly decreased in all treatment groups except plasma AMH levels in the metformin group when compared to the PCOS group (*p* < 0,001). No significant difference was observed between treatment groups. Tissue and plasma AMH levels were in accordance with each other.

#### TNF- ɑ - an inflammation marker

TNF-ɑ levels were significantly higher in the PCOS group than in the control group (*p* < 0,05) and all treatments significantly reduce these hormone levels compared to the PCOS group (*p* < 0,05). No significant difference was observed between treatment groups. Tissue and plasma TNF- ɑ levels were correlated with each other.

#### MDA - lipid peroxidation marker

MDA levels were significantly higher in the PCOS group than in the control group (*p* < 0,05). When compared with the PCOS group, the treatment groups significantly reduced serum MDA levels (*p* < 0,05) except the metformin group. No significant difference was observed between treatment groups.

### Light microscopy

**Fig. 1:**
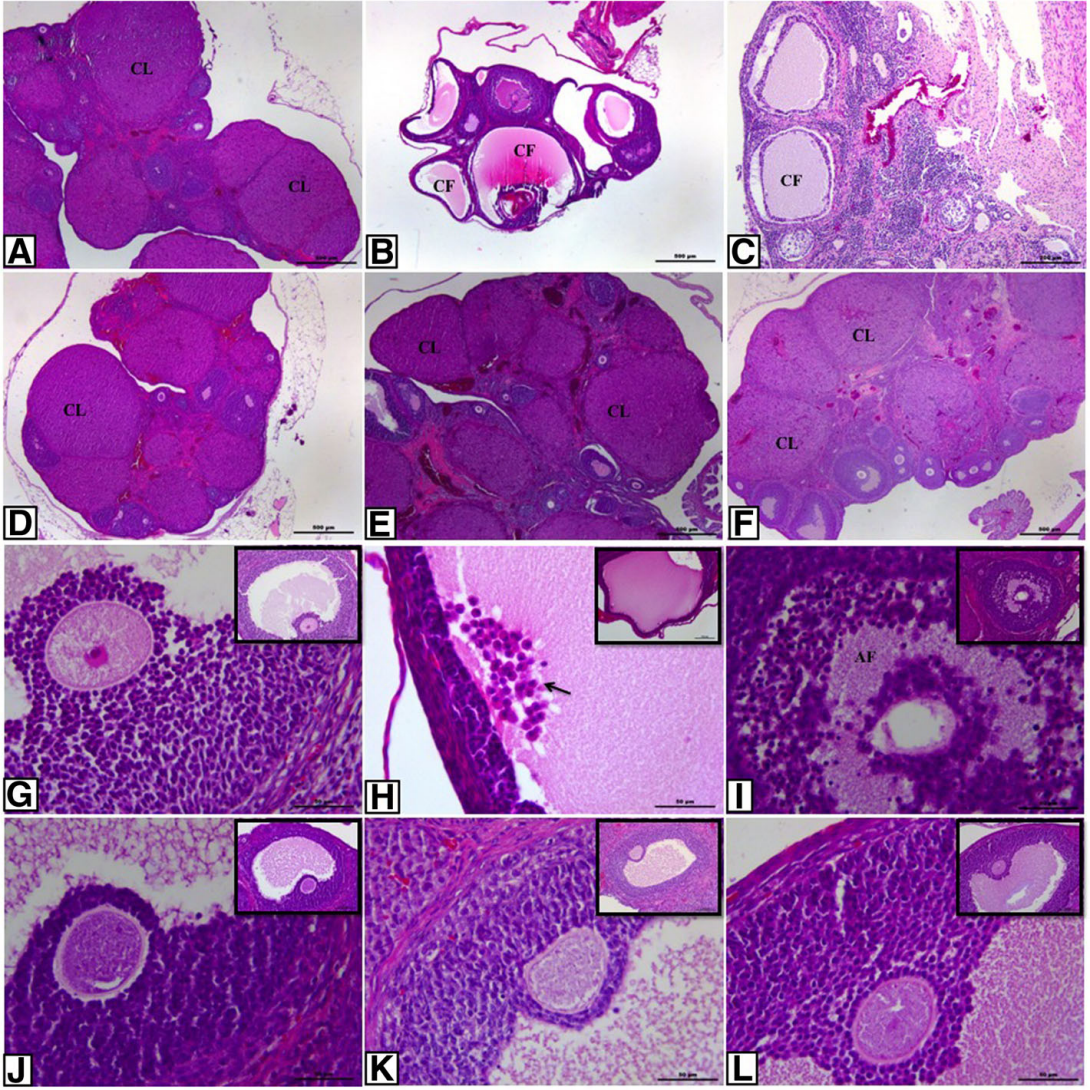
Histopathological alterations in ovarian sections of the control and the experimental groups. **a** control; **b** PCOS; **c** solvent; **d** resveratrol; **e** metformin and **f** metformin+resveratrol group. **g**-**l** higher magnification of the follicles in these groups, respectively. Control group exhibited normal ovarian morphology whereas PCOS and solvent groups showed many cystic and atretic follicles. Treatment groups improved ovarian architecture as indicated by increasing number of corpus luteum and Graafian follicle. CF indicates cystic follicle; AF, atretic follicle; CL, corpus luteum; arrow, apoptotic granulosa cells in antrum. (**A**-**F**, 4X magnification; **G**-**L**, 40X magnification, H&E)

Ovarian sections of the control group exhibited normal ovarian morphology and all types of follicles at different stages of maturation and also corpus luteum which is an indicator of ovulation. Granulosa and theca cells displayed regular and intact organisation. Oocyte and surrounding zona pellucida showed no degeneration (Fig. 1a and g). General morphology of the PCOS and solvent groups displayed lots of cystic and atretic follicles and a few corpus luteum. Granulosa cell layers were decreased and loosely arranged. Moreover, there were seen many granulosa cells in cystic follicles’ full-filled antrum. Apoptotic granulosa cells with pycnotic nuclei, which indicate atresia of follicle, were especially seen in secondary follicles in PCOS groups. Degenerating oocyte and zona pellucida were also seen (Fig. 1b, c, h, i). In the treatment groups, ovarian architecture was improved to comparable levels with the control group. Oocyte and surrounded zona pellucida of follicles were normal and intact (Fig. 1d-f and j-l).

In addition to morphological findings, follicle counts were analyzed to examine different types of follicles in folliculogenesis (Table 3). Primordial follicle pool and Graafian follicles were significantly diminished in the PCOS group compared to the control group (*p* < 0,05), but the number of these follicles in the treatment groups were significantly higher in the PCOS group (*p* < 0,05). However, no significant difference was observed between treatment groups. Unilaminar and multilaminar primary follicle population significantly decreased in all groups compared to the control group (*p* < 0,001); no statistical difference was observed between treatment groups and PCOS group. Number of secondary and atretic follicles were elevated in the PCOS group compared to the control group (*p* < 0,001) and all treatments decreased these follicle counts (*p* < 0,05 and *p* < 0,001, respectively); the treatment groups showed no significant difference with each other. Corpus luteum was significantly decreased in the PCOS group compared to the control group (*p* <0.001) whereas remarkably increased in the treatment groups compared to the PCOS group (*p* < 0,05). The treatment groups did not differ in terms of number of corpus luteum as well.

**Table 3:** Mean ± SEM number of ovarian follicles in control, PCOS, resveratrol, metformin and metformin+resveratrol treated rats

Parameters	Control	PCOS	Resveratrol	Metformin	Metformin+Resveratrol	%5 Ethanol	Saline
Primordial follicle	75 ± 2.88	20.64 ± 2.68^**^	56.56 ± 2.86^+^	56.67 ± 5.01^+^	49.56 ± 4.70^+^	27.86 ± 4.33^*^	30.43 ± 4.64^*^
Unilaminar primary follicle	51.57 ± 6.72	33.73 ± 1.63^**^	32.78 ± 2.01^**^	32.89 ± 0.42^**^	32.67 ± 0.53^**^	31.71 ± 1.63^**^	32.29 ± 5.13^**^
Multilaminar primary follicle	40.14 ± 0.51	21.36 ± 1.50^**^	20.78 ± 0.95^**^	20.11 ± 0.95^**^	20.67 ± 0.78^**^	24.57 ± 0.75^**^	25.71 ± 1.02^**^
Secondary follicle	9.29 ± 0.47	13.09 ± 0.34^**^	9.56 ± 0.44^+^	9.89 ± 0.42^+^	9.56 ± 0.58^+^	12.86 ± 0.4^**^	12.86 ± 0.63^**^
Graafian follicle	7.57 ± 0.65	1.73 ± 0.38^**^	5.44 ± 0.56^+^	5.11 ± 0.87^+^	6 ± 0.96^+^	2.57 ± 0.97^**^	1.86 ± 0.63^**^
Corpus luteum	13 ± 1.45	2 ± 0.36^**^	13.22 ± 1.53^++^	13.22 ± 1.38^++^	14.89 ± 1.18^++^	3.57 ± 0.37^**^	4.29 ± 0.42^**^
Atretic follicle	1.43 ± 0.3	6.09 ± 0.34^**^	1.78 ± 0.32^++^	2.67 ± 0.33^++^	2.56 ± 0.44^++^	5.43 ± 0.65^**^	5.14 ± 0.34^**^

Values are expressed as mean ± SEM

*, ** versus control group; P < 0,05 and P < 0.001, respectively

+, ++ versus PCOS group; P < 0,05 and P < 0.001, respectively

### Immunohistochemistry

#### SIRT1 immunoreactivity

**Fig. 2:**
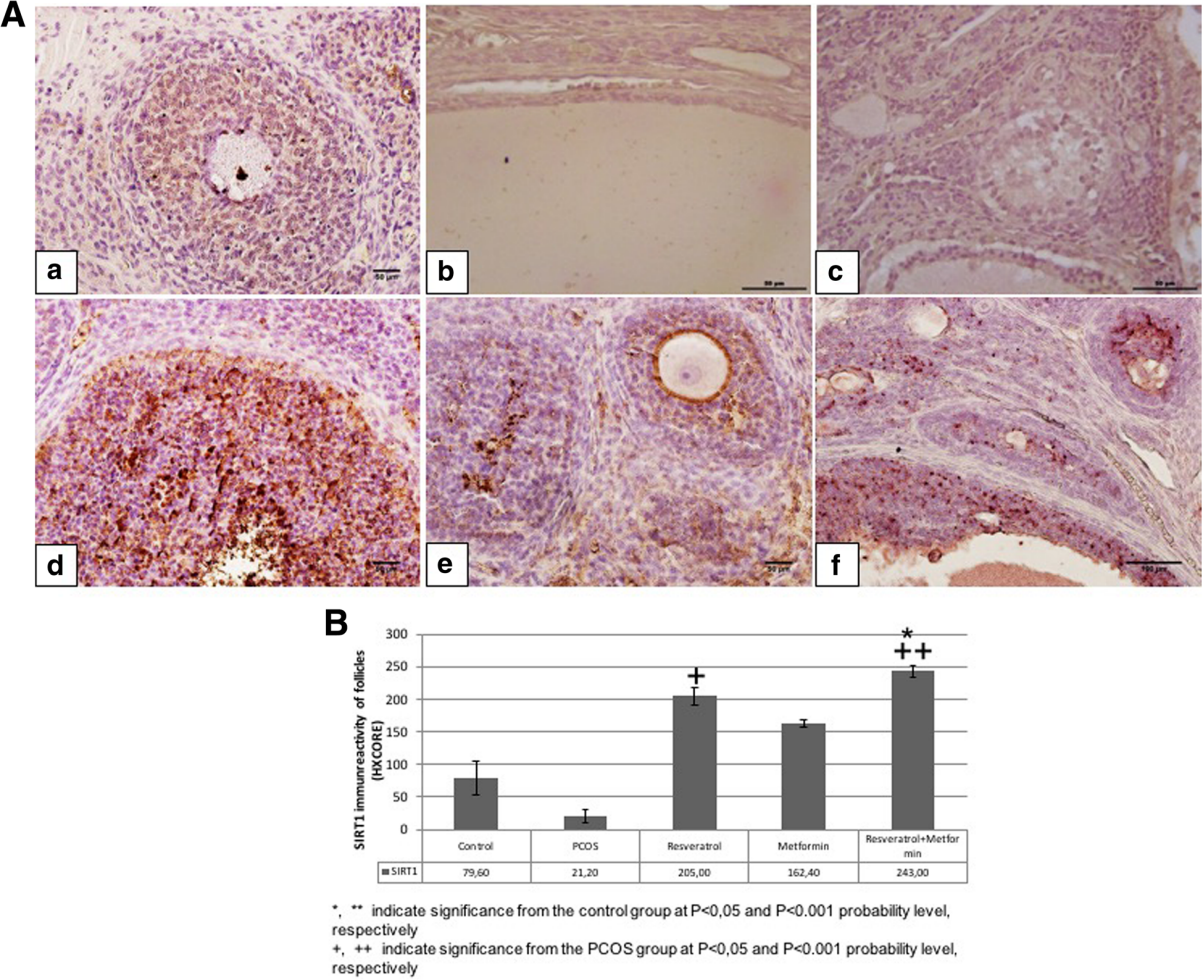
**A** Representative SIRT1 immunreactivity in control, PCOS, resveratrol, metformin and metformin+resveratrol groups. **a**; control, **b**; PCOS, **c**; solvent, **d**; resveratrol, **e**; metformin and **f**; metformin+resveratrol groups. **B** HSCORE of SIRT1 immunostaining in control, PCOS, resveratrol, metformin and metformin+resveratrol groups

SIRT1 immunoreactivity in ovaries belonging to the control and experimental groups were examined (Fig. 2). Immunohistochemical SIRT1 staining intensity in the nucleus of all follicular cells were evaluated using semiquantitative HSCORE method. No significant difference was observed between PCOS group compared to control group. SIRT1 expression was significantly higher in the resveratrol and metformin+resveratrol group compared to the PCOS group (*p* < 0.05 and *p* < 0.001, respectively). The treatment groups did not differ significantly in terms of SIRT1 expression among themselves.

#### AMPK immunoreactivity

**Fig. 3:**
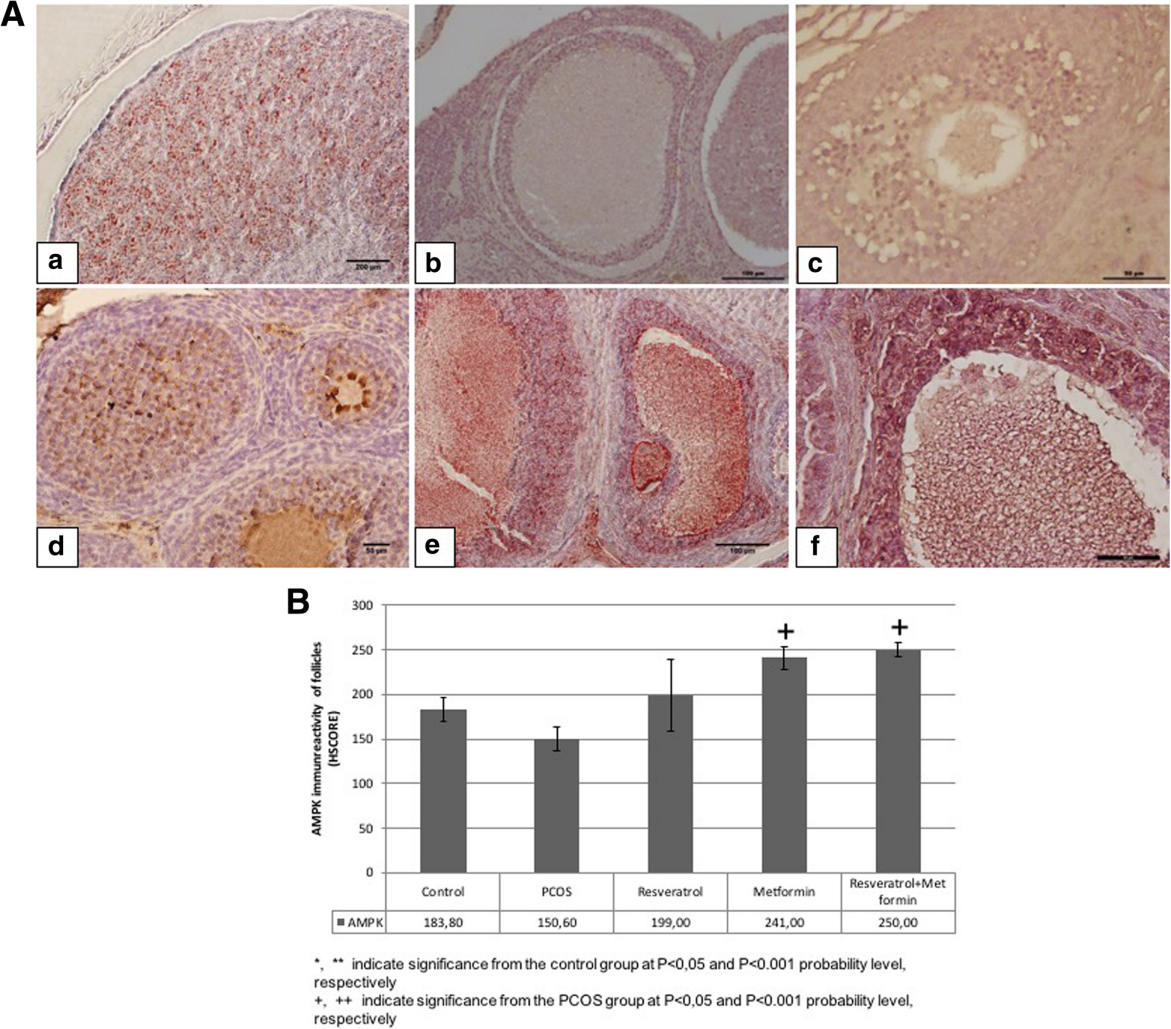
**A** Representative AMPK immunreactivity in control, PCOS, resveratrol, metformin and metformin+resveratrol groups. **a**; control, **b**; PCOS, **c**; solvent, **d**; resveratrol, **e**; metformin and **f**; metformin+resveratrol groups. **B** HSCORE of AMPK immunostaining in control, PCOS, resveratrol, metformin and metformin+resveratrol groups

AMPK immunoreactivity in ovaries belonging to the control and experimental groups were examined (Fig. 3). Immunohistochemical AMPK staining intensity of all follicles were evaluated using semiquantitative HSCORE method. No significant difference was observed between control, PCOS and resveratrol groups in terms of AMPK immunreactivity. AMPK expression were significantly higher in the metformin and metformin+resveratrol group than in the PCOS group (*p* < 0.05). The treatment groups did not differ significantly in terms of AMPK expression among themselves.

#### TUNEL

**Fig. 4:**
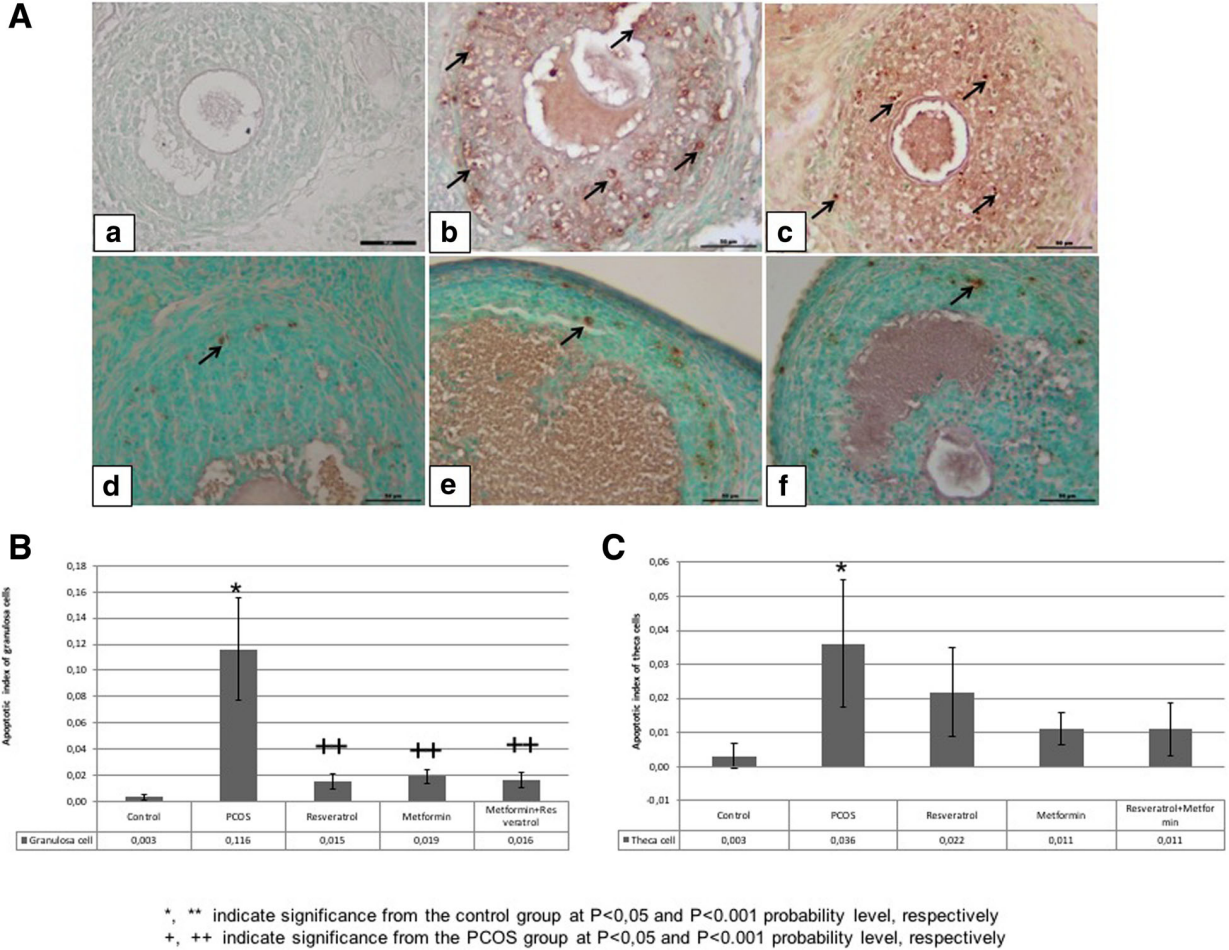
**A** TUNEL analysis of granulosa and theca cells in control, PCOS, resveratrol, metformin and metformin+resveratrol groups. **a**; control, **b**; PCOS, **c**; solvent, **d**; resveratrol, **e**; metformin and **f**; metformin+resveratrol groups. Apoptotic cells are indicated as brown and shown with black arrows. **B** Apoptotic index of TUNEL (+) granulosa and theca cells in control PCOS, resveratrol, metformin and metformin+resveratrol -treated rats’ follicles

In ovarian follicles belonging to the control and experimental groups, apoptotic cells in granulosa and theca cell layer were evaluated by TUNEL method and apoptotic index was calculated (Fig. 4). Apoptotic index of granulosa cells of the follicles were significantly increased in the PCOS group compared to the control group (*p* < 0,05). It was noticed that the PCOS-induced apoptosis were mostly seen in the granulosa cells of Graafian follicles. Decreasing number of TUNEL (+) granulosa cells in the treatment groups were statistically significant compared to the PCOS group (p < 0,001). TUNEL (+) cells in theca cells of PCOS group increased significantly compared to the control group (p < 0,05). No significant difference was observed between PCOS and treatment groups regarding apoptotic indices in these cells. The treatment groups did not show any significant difference with each other.

### Electron microscopy

**Fig. 5:**
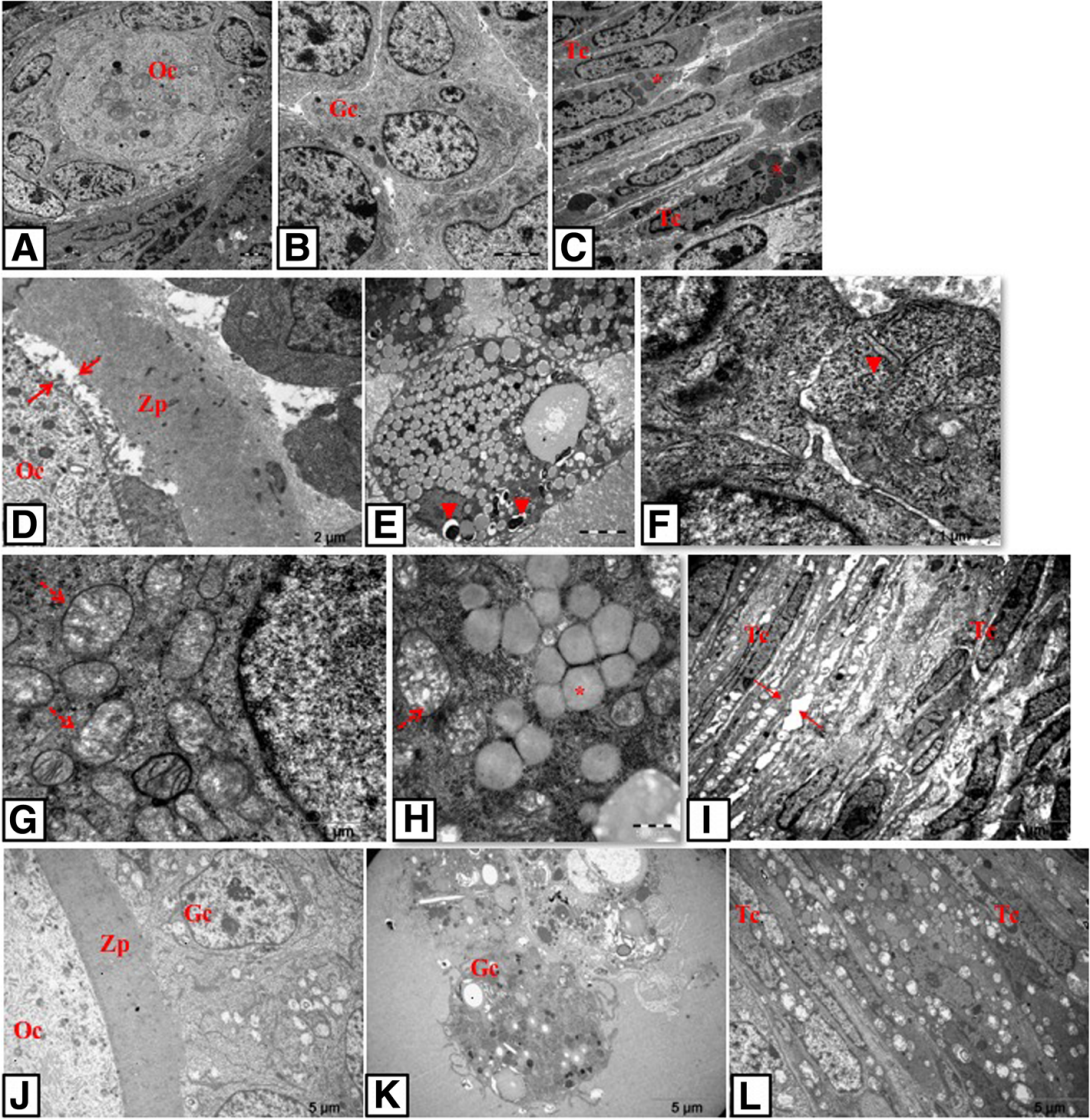
TEM photomicrographs of control and PCOS groups. Oocyte, granulosa and theca cells of follicles in control group (**a-c**), in PCOS group (**d-i**) and in solvent group (**j-l**). D shows the relation between oocyte-zona pellucida-granulosa cells; **e-h** show granulosa cells and I shows the theca cell layer. Note the apoptotic granulosa (**e**, **k**) and theca cells (**i**, **l**) in PCOS and solvent groups. Arrows indicate expanded perivitellin (**d**) and intercellular spaces (**d**, **i**); arrowheads indicate expanded sER (**f**) and autophagosomes (**e**); *: lipid droplets; dashed arrows (**g**, **h**): mitochondria with degenerated cristae. Oc: oocyte, Gc: granulosa cell, Tc: theca cell, Zp: zona pellucida. Uranyl acetate and lead citrate

When the ultrastructures of follicles were examined in the control group, oolemma surrounding the oocytes were uniform and integral with the adjacent follicular cells and in the cytoplasm of the oocytes were the scattered mitochondria. Granulosa cells displayed euchromatic nuclei containing prominent nucleolus and mitochondria with intact cristae and uniform membrane structure. Theca cells exhibited typical steroid-expressing cell characteristics with lipid droplet content in the follicles (Fig. 5a-c).

In the PCOS group, degeneration in zona pellucida and enlargement of perivitellin space were observed (Fig. 5d). A large number of apoptotic granulosa cells spilling in the antrum were seen in PCOS and solvent groups (Fig. 5e, k). These cells exhibited enlarged sER (Fig. 5g), swollen and degenerated mitochondria (Fig. 5h-i), increased number of autophagosomes (Fig.5e, j, k), and excessive lipid droplets (Fig. 5f). Furthermore, expanded intercellular spaces between granulosa cells were observed in these groups (Fig. 5e, g, k). In theca cell layer, an increase in intercellular space, large amounts of degenerated mitochondria with deteriorated cristae structure, excessive lipid accumulation, and abundant autophagosomes were detected (Fig. 5f, l).

**Fig. 6:**
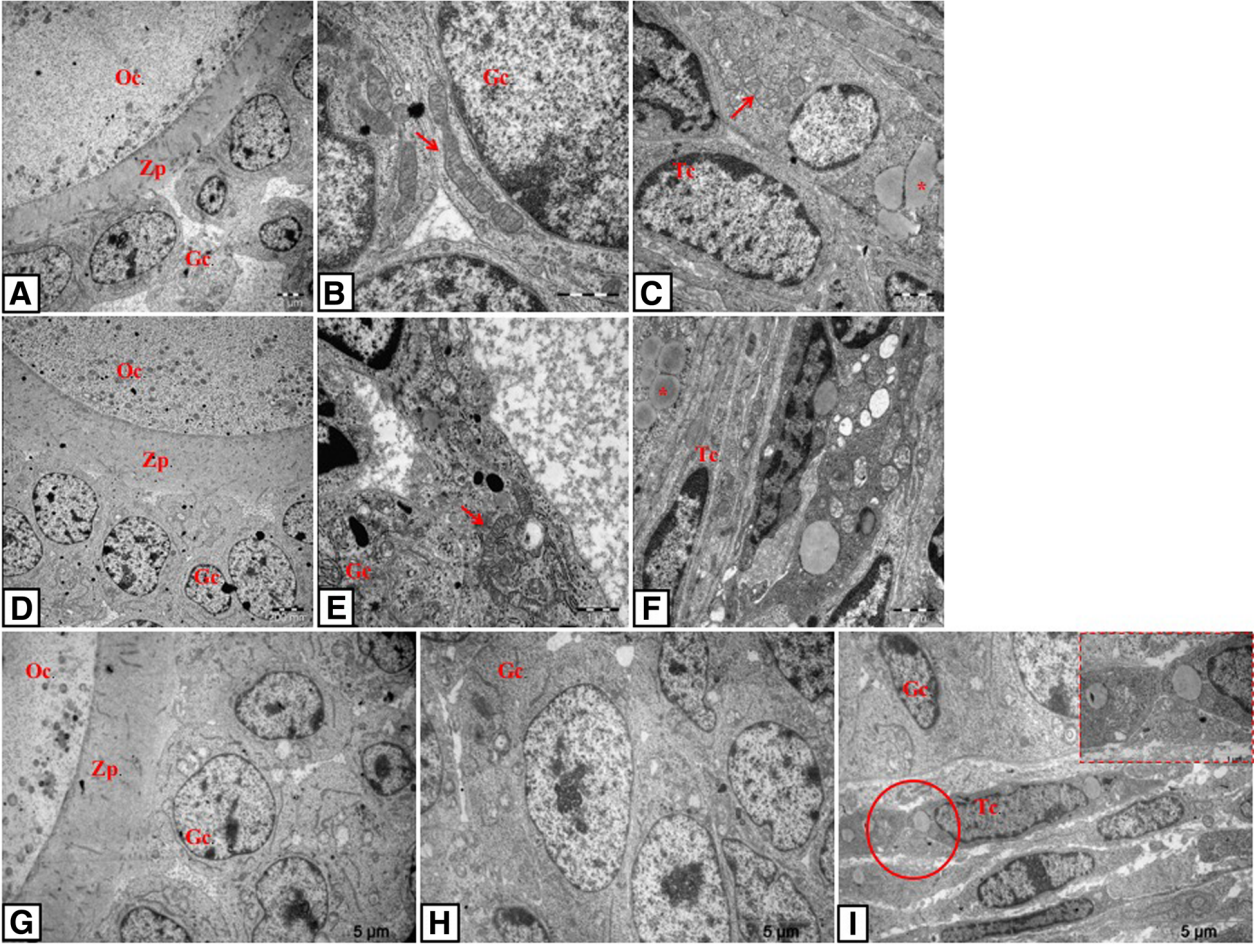
TEM photomicrographs of resveratrol, metformin and metformin+resveratrol-treated rats. Oocyte, granulosa and theca cells of follicles in resveratrol group (**a-c**), in metformin group (**d-f**) and in metformin+resveratrol group (**g-i**). Arrows indicate healthy mitochondria with normal cristae, *: lipid droplets. Oc: oocyte, Gc: granulosa cell, Tc: theca cell and Zp: zona pellucida. Uranyl acetate and lead citrate

When oocytes belonging to the treatment groups were examined ultrastructurally, intact zona pellucida structure and integrity between the surrounding granulosa cells were determined (Fig. 6a, d, g). Follicular cells of the treatment groups were observed to contain less degenerated mitochondria, autophagosomes and lipid droplets and the follicles in these groups had less apoptotic granulosa cells than those of the PCOS group. Healthy mitochondria with cristae structure were detected in the granulosa cells (Fig. 6b, e). It was also found in these cells that the sER structure reversed to normal morphology (Fig. 6e). Regular arrangements of theca cell layer and healthy mitochondrial structure in these cells were also observed (Fig. 6c, f, i).

## Discussion

PCOS has been regarded as a chronic systemic disease, therefore in vitro studies are not enough to explain its pathogenesis mechanism exactly and should be supported by in vivo studies. For this reason we established our study as an in vivo rat modeling. We aimed to investigate the potential therapeutic effects of metformin and resveratrol against the PCOS-induced ovarian damage in terms of biochemical, histological, immunohistochemical, and ultrastructural aspects.

Various treatments such as androgens, estrogens, aromatase inhibitors, antiprogestins, changes in light exposure, and genetic manipulations have been described to establish rodent PCOS models. However, hyperandrogenism is the most consistent PCOS feature and hence most recent studies have focused on using androgens to induce PCOS in rodent models and they have clearly showed that excess androgen can induce many reproductive and metabolic features of human PCOS [55]. DHEA is considered to be the key agent in androgen biosynthesis and known to effect polycystic changes in the rat ovary [56]. Studies with DHEA-treated postnatal mice and rats resulted in anovulation, follicular cysts with a thin granulosa cell layer, increased numbers of atretic follicles, increase in fat and stroma in ovaries, altered ovarian steroidogenesis with elevated serum levels of androgens, estrogens, P, and prostaglandin [55]. In other studies, it was found that DHEA-induced hyperandrogenism increases ovarian lipid peroxidation and decreases catalase activity and glutathione content whereas prepubertal hyperandrogenism increases serum TNF-α levels and consequently increasing lipid peroxidation and impairing ovarian function [57]. Although the effects of exogenous androgens on ovarian morphology may vary depending on the period and duration of the androgen administration and the amount of hormone administered, we established our PCOS model with DHEA based on the previous studies.

Since hyperandrogenism plays a major role in the pathogenesis of PCOS, we used DHEA to induce PCOS modelling [55, 56]. To prove the anovulation; we monitored the rats’s estrus cycle and make sure that they lost regular estrous cycle as shown previously [49]. Increased LH/FSH ratio and AMH levels in PCOS group also supported our successful modelling.

In this study, metformin alone or combined with resveratrol reduced the augmented body and ovary weights in the PCOS group. Metformin’s weight reducing effects were reported previously and associated with its inducing effect on intracellular AMPK. AMPK reduces the activity of acetyl CoA carboxylase enzyme (ACC) and low activity of ACC decreases the biosynthesis of fatty acids and then increases β-oxidation, which can promote weight loss [58, 59]. Our AMPK immunostaining data support this relation between AMPK and weight reducing effect of metformin. Although resveratrol could not significantly reduce body weight alone, based on SIRT1 immunostaining findings we suggest that its ovarian weight reducing effect might be mediated by SIRT1 activation. SIRT1 is a target which acts through resveratrol and helps in increased oxidative phosphorylation, fatty acid oxidation, reduction in fatty acid synthesis and stimulate lipolysis [60].

Serum testosterone levels were higher in DHEA-induced PCOS rats but these levels were significantly decreased in all treatment groups compared to PCOS group. This decrease might be a result of anti-proliferative or pro-apoptotic effects of resveratrol on theca-interstitial cells as shown by previous studies [61, 62], although our TUNEL assay results regarding theca cells are not in accordance with these data. However, our ultrastructural findings related to theca cells exhibited that mitochondrial damage and expanded sER were ameliorated in the resveratrol group. Resveratrol alone completely reversed the testesterone levels to the control levels, whereas testosterone levels in the metformin alone and combined therapy group were higher compared to the control group. Thus, it can be inferred from our results that resveratrol plays a more effective role in reducing testosterone levels. In parallel to our findings, it has been reported that serine-threonine kinase/protein kinase B pathway is a cell-signaling pathway included in ovarian steroidogenesis and its activity was decreased by resveratrol administration [63]. On the other hand, metformin’s suppressive effects on ovarian steroidogenesis were described previously [32, 33]. We suggest that resveratrol and metformin may also regulate ovulation by acting on gonadotropin hormones, since LH levels were significantly lower in the treatment groups than in the PCOS group. As a phytoestrogen, resveratrol may regulate HPG axis via affecting gene transcription in estrogen-sensitive tissues [64].

DHEA-induced PCOS diminished the primordial follicle pool, however, primary follicles were not as much as expected in the PCOS group. Additionally, secondary and atretic follicles were increased while Graafian follicles were decreased in the PCOS group which is attributed to the cessation of folliculogenesis [11]. Since secondary and atretic follicle numbers were elevated in the PCOS group, we suggest that some of the primary follicles have turned into secondary follicles and some have suffered from atresia. In parallel to the increase in secondary follicle numbers, higher AMH levels were detected in the PCOS group. In a previous study high serum and intrafollicular levels of AMH in PCOS patients has been reported due to increased number of small antral follicles. This excess of AMH plays a role in the characteristic follicular arrest of PCOS via inhibition of aromatase expression and FSH action [65]. Since estrogen is known to be a survival factor for the maintainance of granulosa cells, inhibition of aromatase results in apoptosis of granulosa cells due to lower conversion of androgen to estrogen [66]. Therefore, we suggest that AMH induces the follicular atresia via indirectly inhibiting estrogen secretion. This hypothesis was supported by increased number of TUNEL (+) granulosa cells and atretic follicles in the PCOS group in our study. In this study, treatment with resveratrol and/or metformin ameliorated the follicle numbers, which indicates the effect of the treatments on the maintenance of folliculogenesis. Decreasing number of secondary follicles in the treatment groups are consistent with decreasing levels of AMH. We demonstrated that higher plasma AMH levels in PCOS rats were improved by resveratrol, but not metformin. This reducing effect of resveratrol has been demonstrated before by Ergenoglu et al. [67], however they have administered a different dose of resveratrol to DHT-induced PCOS rats. Resveratrol, acting through its AMH decreasing effect, may activate aromatase expression and eventually support follicullogenesis.

OS is considered as an inducing factor in the pathogenesis of PCOS and circulating markers of oxidative status, such as MDA, superoxide dismutase (SOD), and glutathione peroxidase (GPx) were demonstrated to be altered in patients with PCOS [2, 68]. On the other hand, antioxidant effects of resveratrol both in ovary and other tissues have been reported previously [69, 70]. In our study, high levels of MDA, the indicator of lipid peroxidation, observed in the PCOS group were recovered by resveratrol alone and combined therapy, but not by metformin alone which suggests that the improvement of serum MDA levels are due to the antioxidant effect of resveratrol which is attributed to its ROS production reducing properties [23].

The pathogenesis mechanism and etiology of PCOS are complex and it was suggested that inflammation may also be involved in the development of metabolic aberrations and ovarian dysfunction [71]. Previous studies have demonstrated high levels of inflammation markers such as TNF-α and IL-6 in patients with PCOS [72]. Our finding regarding TNF-α is in accordance with the previous data. Resveratrol has been reported as a potent antiinflammatory compound activating AMPK-SIRT1 pathway [36] and it has been suggested to supress TNF-α-induced IL-8 release from the endometrial stromal cells via SIRT1 pathway in endometriosis [73]. In parallel with this data, our results showed for the first time that resveratrol treatment decreased plasma and tissue TNF-ɑ levels in rats with DHEA-induced PCOS. We suggest from our findings that resveratrol may reduce inflammation via SIRT1 activation, since increased SIRT1 immunreactivity was detected in the resveratrol and combined treatment groups. Metformin also seems to have anti-inflammatory effects on women with PCOS as evidenced by a significant decrease in circulating levels of C-reactive protein and white blood cells [74]. Our data also supported this effect of metformin with the finding of reduced TNF-α levels in Metformin-treated rats compared to PCOS group and in addition suggests that this effect may be mediated through AMPK activation since AMPK immunreactivity was higher in the metformin and combined treatment groups compared to the PCOS group. Since resveratrol and metformin are activators of SIRT1 and AMPK, respectively, and since we didn’t set a waiting period after the treatments (sacrification was done just after the treatments), overexpression of SIRT1 and AMPK at the end of the experiment in the treatment groups compared to the control group is acceptable. Based on previous reports regarding the effect of resveratrol and/or metformin on PCOS [14, 67] and biochemical, morphological and ultrastructural results of this study, we suggest that resveratrol and/or metformin may be used to improve ovarian functions in PCOS. Physiological safety of overexpression of SIRT1 and AMPK may be assessed with a multidisciplinary approach if considered necessary.

In vitro studies on the apoptotic effects of resveratrol on different cell types in the ovary have yielded contradictory results. Specifically, in granulosa cells, resveratrol decreased the activity of caspases 3/7 and slightly increased the cell number in vitro [75], however in theca-interstitial cells, it increased the activity of caspases 3/7, inhibited DNA synthesis and decreased the number of viable cells in theca-interstitial cell culture [61]. In our study, in vivo, we observed that resveratrol decreased the apoptotic index of the granulosa cells, but not the theca cells, supporting the previous data.

There is lack of information concerning ultrastructural examination of PCOS. In this study, mitochondrial disruption with degenerated cristae, expanded sER, and high lipid droplet content were observed in the granulosa and theca cells of follicles in the PCOS rats as shown by previous studies [54, 76]. Since mitochondria are responsible for oxidant-antioxidant balance in the cell, degeneration of this organelle indicates an oxidative stress in our PCOS model, which was supported by the increase in serum MDA levels. Moreover, based on the known relation with apoptotic processes, degeneration of the mitochondria suggests the mitochondria-dependent pathway for the apoptosis displayed by TUNEL findings in the PCOS rats. Impaired sER and large amount of lipid droplets observed in this group might be related to hormonal abnormality. It has been already known that sER is responsible for testosterone synthesis in the cell and as a result of AMH-induced impaired aromatase expression, androgen accumulation may occur in follicular cells in PCOS [77]. So, this accumulation may disrupt sER structure and cause dilatations in the organelle. Increased number of autophagosomes in the granulosa cells was another ultrastructural observation in the PCOS group in this study. Recent studies demonstrate that autophagy was enhanced in the ovarian tissue of both humans and rats with PCOS. Consistent with this, granulosa cells from PCOS rats showed increases in the autophagy marker [78]. All ultrastructural findings were ameliorated by the treatments. In addition to inhibitory effect on steroidogenesis [63], antioxidant capacity of resveratrol has a major role in this improvoment. It has been known that resveratrol effectively scavenges hydroxyls and superoxides and protects against lipid peroxidation in cell membranes and DNA damage caused by ROS [79]. It has been reported that SIRT1 activation inhibits hyperglycemia-induced apoptosis by reducing OS and mitochondrial dysfunction in human endothelial cells [80]. As a result of these data, we infer that resveratrol improved mitochondrial ROS production via SIRT1 activation. Ultrastructural findings also supported the potential therapeutic effects of resveratrol and metformin on granulosa and theca cells of follicles in PCOS group.

## Conclusions

This is the first study to reveal the effects of combined metformin and resveratrol treatment in a rodent model of PCOS. The data of this study revealed that regarding the majority of the findings, there were no significant difference between resveratrol and metformin and resveratrol was superior in ameliorating the PCOS (decreased plasma AMH and serum MDA levels), which indicate that resveratrol may be an alternative to metformin or may be used with low-dose metformin in PCOS treatment. Since patients suffer from gastrointestinal symptoms due to the using of metformin, the next step of this study is to establish dose-dependent experiments in which resveratrol dose is stabilized while metformin’s is reduced. To examine the effects of SIRT1 and AMPK overexpression in the treatment groups, recovery/waiting period after treatments should be added to the study design for the next step planning. Also, other molecules related to this pathway could be investigated with further and more accurate techniques. Thus, the effectiveness of resveratrol can be evaluated preciesely. Although next step in vivo study is necessary, a clinical trial is the only way to determine if resveratrol alone or in combination with metformin will improve PCOS in the human*”.*

## Abbrevations

AEC: 3-amino-9-ethylcarbazole
AMH: Anti mullerian hormone
AMPK: Adenosine monophosphate activated kinase
APSAA: Acute phase serum amyloid A
CRP: C-reactive protein
DAB: Diamino benzidine
DHEA: Dehydroepiandrosterone
FSH: Follicle stimulating hormone
I.p: Intraperitoneally
IL-6: Interleukin-6
LH: Luteinizing hormone
MCP-1: Monocyte chemotactic protein-1
MDA: Malondialdehyde
OS: Oxidative stress
PBS: Phosphate buffered saline
PCOS: Poly cystic ovary syndrome
ROS: Reactive oxygen species
S.c: Subcutaneously
sER: Smooth endoplasmic reticulum
SIRT1: Silent information regulator 1
SOD: Superoxid dismutase
TNF-α: Tumor necrosis factor-a
